# Microwave Assisted Extraction and Pressurized Liquid Extraction of Sulfated Polysaccharides from *Fucus virsoides* and *Cystoseira barbata*

**DOI:** 10.3390/foods10071481

**Published:** 2021-06-25

**Authors:** Ana Dobrinčić, Sandra Pedisić, Zoran Zorić, Mladenka Jurin, Marin Roje, Rozelindra Čož-Rakovac, Verica Dragović-Uzelac

**Affiliations:** 1 Faculty of Food Technology & Biotechnology, University of Zagreb, Pierottijeva 6, 10 000 Zagreb, Croatia; adobrincic@pbf.hr (A.D.); spedisic@pbf.hr (S.P.); zzoric@pbf.hr (Z.Z.); vdragov@pbf.hr (V.D.-U.); 2 Ruđer Bošković Institute, Biljenička cesta, 10 000 Zagreb, Croatia; mladenka.jurin@irb.hr (M.J.); Rozelindra.Coz-Rakovac@irb.hr (R.Č.-R.)

**Keywords:** *Fucus virsoides*, *Cystoseira barbata*, polysaccharides, fucoidan, microwave assisted extraction, pressurized liquid extraction

## Abstract

Sulfated polysaccharide fucoidan isolated from brown algae shows a wide range of biological activities that are significantly dependent on its chemical composition, which is closely related to the applied technique and extraction parameters. Therefore, the objective of this study was to evaluate the influence of microwave assisted extraction (MAE) and pressurized liquid extraction (PLE) parameters (solvent, temperature, time, and number of cycles) on the *Fucus virsoides* and *Cystoseira barbata* polysaccharide yield (%PS) and chemical composition (total sugar, fucose, and sulfate group). The optimal MAE parameters that resulted in the highest polysaccharide extraction from *F. virsoides* and *C. barbata* were 0.1 M H_2_SO_4_ for 10 min at 80 °C, while the optimal PLE parameters were 0.1 M H_2_SO_4_, for two cycles of 15 min at 140 °C. Furthermore, the %PS, chemical structure, molecular properties, and antioxidant activity of the *F. virsoides* and *C. barbata* polysaccharide extracts obtained with MAE, PLE, and conventional extraction (CE) performed under previously determinate optimal conditions were compared. PLE resulted in a significantly higher %PS from *F. virsoides,* while for *C. barbata*, a similar yield was achieved with CE and PLE, as well as CE and MAE, for both algae. Furthermore, the polysaccharides obtained using PLE had the highest polydispersity index, fucose, and sulfate group content, and the lowest uronic acid content; however their antioxidant activity was lower.

## 1. Introduction

Because of the increased consumer awareness of functional food ingredients, seaweeds are increasingly being considered as a potential source of bioactive compounds. With about 2000 species, brown seaweeds are the second most abundant group of marine algae [1]. Their various biological properties, such as their anticoagulant, antithrombotic, anti-viral, anti-cancer, anti-inflammatory, and antibacterial effects [2], have been attributed to the sulfated polysaccharide fucoidan. Fucoidan is composed of mainly fucose interconnected by *β* (1, 3) glycoside bonds; alternating *β* (1, 3) and *β* (1, 4) bonds; and, rarely, *β* (1, 2) bonds [3]. Apart from fucose, it also contains other monosaccharides, including galactose, glucose, mannose, xylose, rhamnose, and uronic acids, and its sulphate content varies between 5% and 38% [3]. The chemical structure of fucoidan may significantly determine its physical, chemical, and biochemical properties [4]. Moreover, the biological activities of fucoidan are strongly associated with their chemical structure [5]; however, the correlation between the structure and biological activity has still not been sufficiently clarified [2]. The structure and composition of fucoidan can be influenced by the algae species, location, and harvesting season [6], as well as extraction techniques and different extraction conditions (e.g., pH, time, temperature, pressure, particle size, solvent, sample to solvent ratio, and agitation speed) [5,7,8].

Conventional polysaccharide extraction (CE) is generally performed with water, dilute acid, or dilute alkali for a long time; at a high temperature; and using a large solvent volume, and is thus not economically and environmentally friendly. To overcome these limitations, advanced technologies such as microwave assisted extraction (MAE), ultrasound assisted extraction (UAE), pressurized liquid extraction (PLE), and enzyme-assisted extractions (EAE) have been applied to extract brown algae polysaccharides (PS). MAE utilizes microwave energy for heating, and thus increases the mass transfer rate of the solutes from the sample matrix into the solvent [9]. The MAE open vessels system under atmospheric pressure can be operated at a maximum temperature determined by the boiling point of the solvents [10]. In PLE, elevated temperatures and pressures are used to extract compounds from samples in an oxygen and light-free environment, in a short period of time and using less solvent [11]. Elevated pressure keeps the solvent below its boiling point so the application of temperatures above a solvent boiling point (at atmospheric pressure) is possible.

The benthic fauna of the Adriatic Sea includes 2597 species of algae, 152 of which are endemic. One of the endemic brown algae species is *Fucus virsoides*, the only representative of Fucus genus in the Mediterranean, growing mainly in the northern Adriatic, from the Venice Lagoon to Dalmatia [12]. *Cystoseira barbata* belongs to the genus Cystoseira, whose representatives have an important role in the structure and functioning of the rocky habitats of the Mediterranean and the Black Sea, providing shelter, food, and nursery grounds for a variety of organisms [13].

Even though MAE and PLE have been successfully applied for the extraction of numerous biologically active compounds from a wide variety of plants, their application in brown algae PS extraction is sparsely reported [1,14,15], especially their comparison in terms of the yield and chemical composition of the extracted PS. As conventional PS extraction is long and complex, a significant time reduction and lower energy consumption makes these novel techniques really interesting for application in processed and functional food, pharmaceutical, and chemical industries. In this research, MAE and PLE were applied to extract polysaccharides from brown algae *F. virsoides* and *C. barbata,* and one of the goals was to investigate the influence of extraction parameters (solvent, time, temperature, and number of cycles) on the yield and chemical structure (total sugar, fucose, and sulfate group content) of the extracted PS. Furthermore, the yield, chemical structure (total sugar, fucose, sulfate group and uronic acid content, and monosaccharide composition), molecular weight, and antioxidant activity of the PS extracts obtained with MAE, PLE, and CE, performed under previously determined optimal conditions [16], were compared.

## 2. Materials and Methods

All of the chemicals and reagents used in this study were of analytical grade. Ethanol, acetone, sodium carbonate (Na_2_CO_3_), and potassium sulfate (K_2_SO_4_) were purchased from Gram-mol doo (Zagreb, Croatia); sodium tetraborate, L-cystein, sulfamic acid, gelatin, potassium hydroxide, chloroform, 1-phenyl-3-methyl-5-pyrazolone (PMP), and 6-hydroxy-2,5,7,8-tetramethylchroman-2-carboxylic acid (Trolox) from Acros Organics (Geel, Belgium); acetonitrile, trichloroacetic acid (TCA), silicone oil, and Folin–Ciocalteu reagent from Fisher Scientific (Leicestershire, UK); barium chlorid (BaCl_2_) from abcr GmbH (Karlsruhe, Germany), fucoidan from *Fucus vesiculosus*, D-galacturonic acid, phenol, *m*-hydroxydiphenyl, 2,2-diphenyl-1-picrylhydrazyl (DPPH), arabinose, D-(+)-glucose, L-rhamnose, D-(−)-fructose, trimethylamine, 2,2′-Azobis(2-methylpropionamidine) dihydrochloride (AAPH), D-(+)-mannose, L-(−)-fucose, and ammonium acetate from Sigma-Aldrich (St. Louis, Missouri, USA); ethyl acetate, hexane, and absolute ethanol from Carlo Erba Reagents (Cornaredo, Italy); hydrochloric acid (HCl) from TKI Hrastnik (Hrastnik, Slovenia); sodium hydroxide from Lach-Ner (Zagreb, Croatia); sulfuric acid (H_2_SO_4_) from Scharlab S.L. (Barcelona, Spain); D-(−)-ribose from TCI (Portland, OR, USA); and fluorescein sodium salt from Honeywell Riedel-de-Haën (Bucharest, Romania).

### 2.1. Algal Material and Preliminary Treatments

*Cystoseira barbata* was harvested from the coastal region of Zadar, Croatia (44°12′42″ N; 15°09′23″ E), and *Fucus virsoides* was harvested from the southwest coast of the Novigrad Sea, Croatia (44°12′02″ N; 15°28′51″ E), in December 2018, and the identification was performed by marine biologist Donat Petricioli. Freshly collected algae were firstly washed in seawater and then distilled water, and were then frozen at −60 °C in a ScanCool SCL210P freezer (Labogene ApS, Hillerød, Denmark); lyophilisation was done on a CoolSafe lyophilizer, Model: 55-9 PRO, (company, Labogene, Denmark) for 24 h. The lyophilised algae were ground in an electric grinder and were stored at −20 °C until extraction.

### 2.2. Pre-Treatment

The pre-treatment process of the algae samples was performed with continuous stirring in two phases: 18 h at room temperature with acetone, followed by 4 h at 70 °C with 96% ethanol [16]. Afterwards, the residual algae were dried and subjected to conventional, MAE, or PLE PS extraction.

### 2.3. Extraction of Polysaccharides

#### 2.3.1. Conventional Extraction

The pre-treated dried seaweed (1 g) was extracted under constant stirring (400 rpm) at previously determined optimal conditions [16], as follows: 0.1 M H_2_SO_4_ (30 mL) as a solvent, for 3 h at 80 °C.

#### 2.3.2. Microwave Assisted Extraction

The pre-treated dried seaweed (1 g), 30 mL of extraction solvent (H_2_O, 0.1 M HCl, or 0.1 M H_2_SO_4_), and the magnetic stirrer were put into an extraction cell and placed in a microwave reactor, Ethos Easy (Milestone, Italy). The time required to achieve the extraction temperature was set at 2 min; ventilation after extraction at 5 min; extraction temperatures of 60, 80, or 100 °C; and extraction times of 10, 20, or 30 min.

#### 2.3.3. Pressurized Liquid Extraction

PLE was performed using an accelerated solvent extractor (ASE 350, Dionex, Sunnyvale, CA, USA), equipped with a solvent controller. The 22-mL stainless steel extraction cells were filled in consecutive layers with two glass filters on the bottom, with about a 2 cm layer of diatomaceous earth, 1 g of pre-treated dry algae powder mixed with 2 g of diatomaceous earth, and diatomaceous earth again layered on top to fill the extraction cells. Distilled water and 0.1 M H_2_SO_4_ were used as the extracting solvent and a pressure of 1500 psi was retained for all of the analyses. Extractions were performed at different extraction temperatures (60, 100, and 140 °C) in one or two extraction cycles for 5, 10, and 15 min. The warming-up time changed depending on the extraction temperature (2 min if the extraction temperature was 60 °C, 5 min if the extraction temperature was 100 °C, and 6 min if the extraction temperature was 140 °C). The solvent was purged from the cell with nitrogen for 120 s and the system was depressurized. The extracts were collected in glass collection vials and afterwards were transferred to an Erlenmeyer flask.

#### 2.3.4. Processes after Extraction

After extraction, the extracts were filtrated and PS precipitation from the supernatant was done overnight, at 4 °C, by adding double the volume of absolute ethanol. After centrifugation at 5500 RPM for 30 min, the PS were dried at room temperature for 48 h, crushed to a fine powder in a mortar and pestle, and stored at −20 °C.

Extractions were performed in duplicate and the PS extraction yield (% PS) was calculated according to Equation (1), where WP is the weight obtained after ethanol precipitation and WA is the algae weight used in each experiment.

(1) $$\%\;\mathrm{PS}=\frac{WP}{WA}\;\times \;100$$

### 2.4. Chemical Composition of Polysaccharides

The total sugar concentration in the PS was determined using the colorimetric phenol-sulfuric acid method, using glucose as the standard (Dubois et al., [17]). The content of L-fucose units in the PS was determined using a colorimetric assay with L-cysteine, as described by Dische and Shettles [18], using L-fucose as the standard. The sulfate group content was quantified after a hydrolysis of PS in 1 M HCl at 105 °C for 5 h, according to the turbidimetric BaCl_2_-gelatin method, with K_2_SO_4_ as the standard, as described by Dodgson and Price [19]. The uronic acid content was measured with the modified sulfamate/m-hydroxydiphenyl colorimetric method using D-galacturonic acid as the standard, in which sulfamate suppressed the formation of brown pigments from the neutral sugars and tetraborate increased the sensitivity of the reaction with uronic acids (Filisetti-Cozzi and Carpita, [20]). The results were expressed as the percentage of total sugar, fucose, sulfate group, or uronic acids in the dry PS extract.

### 2.5. Monosaccharide Analysis of Polysaccharides by High Performance Liquid Chromatography (HPLC)

The monosaccharide composition was analyzed according to a previously reported method [21], with minor modifications. A sample of PS (100.0 mg) was dissolved in 1 mol/L H_2_SO_4_ (2.0 mL) and was incubated for 4 h at 110 °C. After cooling, the reaction mixture was neutralized to pH 7 with 2 mol/L sodium hydroxide, and the internal standard solution (2 mL) was added. The mixture was shaken well, diluted to 10 mL, and filtered.

The mixture of filtered hydrolyzed PS solution (or monosaccharide standards; 100 μL), 0.5 mol/L methanolic solution of PMP (100 μL), and 0.3 mol/L aqueous sodium hydroxide (100 μL) was incubated at 70 °C for 30 min. The reaction mixture was then cooled and neutralized with 0.3 mol/L hydrochloric acid. Chloroform (1 mL) was added to the solution, shaken well on a vortex and centrifuged at 5000 RPM for 10 min. The chloroform layer was discarded and the aqueous layer was extracted twice with chloroform. The final aqueous layer was analyzed directly by HPLC.

An accurate amount of ribose (~1 mmol) was dissolved in water, diluted to 50 mL, and used as the internal standard solution. Five known concentrations of the following standards (mixed with internal standard) were prepared by consecutive dilutions from stock solutions and were injected into the instrument: glucose (0.25 to 2 mg/mL), fucose (0.1875 to 1.5 mg/mL), galacturonic acid (0.25 to 1.75 mg/mL), arabinose (0.25 to 1.75 mg/mL), mannose (0.25 to 1.75 mg/mL), fructose (0.25 to 1.75 mg/mL), and rhamnose (0.25 to 1.75 mg/mL). Calibration curves (Table 1) were plotted as the ratio of the peak areas of the standard monosaccharide and the internal ribose standard versus concentration.

An HPLC Agilent Infinity 1260 system (Agilent Technologies, Santa Clara, CA, USA) equipped with UV/VIS and DAD, an automatic injector, and ChemStation software on a Zorbax Eclipse XDB-C18 column (4.5 × 250 mm, 5 μm; Agilent Technologies, USA) was used for the analysis. Solvent A was a mixture of 0.4% trimethylamine in 20 mmol/L ammonium acetate buffer solution (pH 6.30 with acetic acid) and acetonitrile in a 9 to 1 ratio. Solvent B was a mixture of 0.4% triethylamine in a 20 mmol/L ammonium acetate buffer solution (pH 6.30 with acetic acid) and acetonitrile in a 4 to 6 ratio. Chromatographic separation was achieved with the following gradient: 0–9 min, 10% to 14% B; 9–30 min, 64% B; 30–35 min, 64% B; and 35–37 min, 10% B. The mobile phase was delivered at a flow rate of 1 mL/min and the column temperature was set at 25 °C. The chromatograms were monitored at 245 nm and the sample injection volume was 10 µL. All of the experiments were carried out in duplicate.

### 2.6. Determination of Molecular Properties

The molecular properties (average molecular weight (M_w_), the number for average molecular weight (M_n_), and the polydispersity index (PDI)) of *F. virsoides* and *C. barbata* PS extracted with CE, MAE, and PLE at optimal conditions were detected and measured using high-performance size exclusion chromatography with a refraction index detector (HPSEC-RID). The 1260 Infinity II LC system (Agilent Technologies) consisted of a quaternary gradient pump G7111B, an autosampler G4767A, a multicolumn thermostat G7116A, and a refraction index detector G7162A. HPSEC analysis was performed using PL aquagel-OH guard column (8 µm, 50 × 7.5 mm; Agilent Technologies) and PL aquagel-OH MIXED-M column (8 μm, 300 × 7.5 mm; Agilent Technologies). The mobile phase was H_2_O, and the temperatures of the column and RID, the flow rate, and the injection volume were set to 30 °C, 40 °C, 0.5 mL/min, and 50 μL, respectively. Data were collected and processed using OpenLAB CDS ChemStation Edition (Agilent Technologies). A molecular weight calibration curve was constructed with known pullulan standards (Agilent Technologies) with M_w_ in a range from 180 to 700 kDa.

### 2.7. DPPH Radical Scavenging Assay

The ability of the extracts to scavenge the DPPH radical was assessed spectrophotometrically. In a test tube, 1.5 mL of polysaccharide solution (1 mg/mL) was mixed with 1.5 mL of DPPH solution (0.2 mM in 70% ethanol), and it was vortexed and kept in the dark at room temperature for 30 min. The decrease in absorbance was measured at 517 nm in duplicate. The free radical scavenging activity was calculated as follows:

Scavenging effect (%) = (A − C)/A × 100(2)

where A is the absorbance of the control (DPPH without sample) and C is the absorbance of the sample.

### 2.8. Oxygen Radical Absorbance Capacity (ORAC) Assay

The antioxidant capacity was evaluated by the oxygen radical absorbance capacity (ORAC) assay according to the research of Elez Garofulić et al. (2020) [22]. The ORAC procedure used an automated plate reader (BMG LABTECH, Offenburg, Germany) with 96-well plates, and the data were analyzed using MARS 2.0 software. Dry PS extracts were dissolved in distilled water (4 mg/mL) and were filtered. AAPH, fluorescein solution, and different dilutions of Trolox were prepared in a 75 μM phosphate buffer (pH 7.4). Dissolved samples were added in a 96-well black plate containing a fluorescein solution (70.3 nM). The plate was incubated for 30 min at 37 °C and after the first three cycles (representing the baseline signal), AAPH (240 mM) was injected into each well to initiate the peroxyl radical generation. On each plate, different dilutions of Trolox (3.12–103.99 μM) were used as the reference standard. The fluorescence intensity (excitation at 485 nm and emission at 528 nm) was monitored every 90 s over a total measurement period of 120 min. The measurements were performed in duplicate, and the results are expressed as μmol of the Trolox equivalents (TE) per g of dry PS sample, as the mean value ± standard deviation (N = 4 replicates).

### 2.9. Statistical Analysis

Statistical analysis was done using STATISTICA v. 8 software (StatSoft Inc., Tulsa, OK, USA). The dependent variables were %PS, total sugar content, fucose content, and sulfate group content, while the independent variables were as follows: (a) solvent (MAE—H_2_O, 0.1 M HCl, and 0.1 M H_2_SO_4_; PLE—H_2_O and 0.1 M H_2_SO_4_), (b) temperature (MAE—60, 80, and 100 °C; PLE—60, 100, and 140 °C), (c) time (MAE—10, 20, and 30 min; PLE—5, 10, and 15 min), and (d) number of cycles in PLE (1 and 2). For a comparison of the different extraction techniques (CE, MAE, and PLE; independent variable), the dependent variables were %PS, total sugar, fucose, sulfate group, uronic acid, and monosaccharide composition. The continuous variables were analyzed using a multivariate analysis of variance (ANOVA). Marginal means were compared with Tukey’s HSD (honestly significant difference) multiple comparison tests. The significance levels for all of the tests were α ≤ 0.05.

## 3. Results and Discussion

### 3.1. Microwave Assisted Extraction

First, it is important to emphasize that all of the extracts analyzed in this study were crude extracts that could contained other co-extracted compounds such as alginic acid [23]. Because the extracts were not purified, we considered it to be more accurate to report their polysaccharide yield (%PS) rather than their fucoidan yield. MAE was applied to extract the PS from *F. virsoides* and *C. barbata,* and the influence of the solvent (H_2_O, 0.1 M HCl, or 0.1 M H_2_SO_4_), temperature (60, 80, or 100 °C), and time (10, 20, or 30 min) on the yield and chemical composition are shown in Table 2. The average crude %PS from *F. virsoides* and *C. barbata* obtained by MAE were 13.19% and 6.43%, respectively. In general, the algae from the *Fucus* genus have reported a PS content in the range of 1.40% to 21.50% [15,24,25,26,27], and the only commercially available source of fucoidan is from *F. vesiculosus*. On the contrary, the reported fucoidan content in the algae from the *Cystoseira* genus ranges from 2.80% to 5.45% [28,29,30,31,32]. Extracts from *F. vesiculosus* obtained by MAE, at a pressure of 120 psi for 1 min and with water as a solvent (solvent-to-sample ratio 25:1 *w*/*v*), had 18.22% sulfated PS [1], while MAE at 120 °C for 30 min with 10 mM sulfuric acid resulted in 11.1% PS from *F. vesiculosus* and 9.52% from *F. serratus* [15]. MAE at 120 °C for 15 min was previously used to extract 16.08% sulfated PS from *Ascophyllum nodosum* [14], while 1000 W of microwave power for 5 min extracted 1699.80 ± 83.80 mg fucose/100 g dw from *A. nodosum* [33]. The optimized MAE conditions of 547 W and 80 °C for 23 min were used to extract 2.84% of PS from brown macroalgae *Sargassum thunbergii* [12]. Furthermore, MAE was likewise used to extract PS from green macroalgae, e.g., at 140 °C for 10 min, the ulvan yield from *Ulva meridionalis* and *Ulva ohnoi* was 40.4 ± 3.2% and 36.5 ± 3.1%, respectively.

The extraction solvent showed a significant (*p* ≤ 0.01) influence on %PS, as both of the applied acids resulted in a significantly higher %PS than water, even three-fold higher with 0.1 M H_2_SO_4_. Cell wall hydrolysis that occurs with the use of acids facilitates PS extraction [34], resulting in a higher %PS with dilute acids compared with the water from brown seaweed *Sargassum fusiforme* [34] and *L. hyperborean* [35]. Additionally, the PS yield increased by lowering the pH [15], as 0.1 M HCl with pH 1 was less effective for PS extraction than 0.1 M H_2_SO_4_ with pH 0.7. Similarly, a better fucoidan and laminarin yield was achieved with 100 mM HCl (pH 2) than with 10 mM H_2_SO_4_ (pH 4) [15].

A shorter extraction time and slightly higher temperature led to an increased %PS. However, an additional increase from 80 to 100 °C did not further improve the extraction yield. Considering these results, the optimal parameters that would result in the highest PS extraction from *F. virsoides* and *C. barbata* were 0.1 M H_2_SO_4_ for 10 min at 80 °C.

This research confirmed that the chemical composition of the extracted PS was influenced by the algal species, extraction solvent, temperature, and time. *F. virsoides* has a higher total sugar and fucose content, but a significantly lower sulfate group content. The average total sugar contents in the PS extracted from *F. virsoides* and *C. barbata* were 15.40% and 6.37%, respectively, which is in accordance with values reported for numerous seaweed species like *C. barbatta, F. vesiculosus*, *C. compressa*, *C. sedoides*, *C. crinite A. nodosum, Sargassum filipendul,* and *Saccharina longicruris,* in which the total sugar content ranged between 8.9% and 66.7% [26,28,29,30,31,36,37,38]. With 58.55% fucose obtained in this research, *F. virsoides* was above the 24–35% range reported for algae from the *Fucus* genus [24], while *C. barbata*, with 26.13% fucose, was within the 16.5–61.5% range for algae from the *Cystoseira* genus [16,30,31]. The *F. virsoides* sulfate group content (25.6%) was within the 9–40.3% range reported for *Fucus* genus [1,24,26,39,40,41], while the 34.8% sulfate groups in *C. barbata* were slightly above the 14.65–22.51% range reported for *Cystoseira* genus [28,29,30,31,32].

The PS obtained with water had a significantly lower (*p* ≤ 0.01) total sugar, fucose, and sulfate group content. The highest total sugar and fucose contents were achieved with 0.1 M HCl, while the sulfate group content was the highest with 0.1 M H_2_SO_4_. Acid promotes sulfate ester breakage, so more sulfate groups can be liberated [23,42], thus explaining the higher sulfate group content obtained by the acids. However, opposite results were reported for *S. fusiforme* [34], *Saccharina japonica* [13], and *Undaria pinnatifida* [43]. Because of the sulfate group in H_2_SO_4_, which might interfere with the sulfate analysis, diluted H_2_SO_4_ tended to yield a high sulfate fucoidan, while diluted HCl tends to yield low sulfate fucoidan [42]; nevertheless, it is generally preferred [23].

A longer time improved the total sugar extraction, while the fucose and sulfate group contents were reduced after 30 min of extraction. A higher temperature resulted in a higher total sugar and lower sulfate group content, but it did not have a significant (*p* ≤ 0.05) influence on the fucose content. The same trend was reported for *A. nodosum* [14] and, similarly, by increasing the pressure from 30 to 120 Psi, which corresponded to a temperature increase from 122 to 172 °C, the *F. vesiculosus* total sugar concentration increased [1].

### 3.2. Pressurised Liquid Extraction

PLE was used to extract the polysaccharides from *F. virsoides* and *C. barbata*, and the influence of the extraction solvent (water and 0.1 M H_2_SO_4_), temperature (60, 100, and 140 °C), time (5, 10, and 15 min), and number of cycles (1 and 2) on the yield and chemical composition of the extracted polysaccharides is shown in Table 3. The average *F. virsoides* %PS obtained by PLE was 10.22%, which is significantly lower than the 11.7% obtained for *C. barbata*. PLE was used to extract fucoidan from *Nizamuddinia zanardinii,* and the obtained yield ranged from 4.99 to 23.77% [44] and 13.15% [45]. Likewise, *S. japonica* crude fucoidan yield obtained with PLE ranged from 0.1 to 12.89% [46] and 8.23% [13].

The type of solvent had the same effect as in MAE on %PS, as the acid lead to a significantly higher %PS for both algae. By increasing the time, temperature, and number of cycles, the *F. virsoides* and *C. barbata* %PS increased. The increased yield obtained at elevated temperatures is explained by the increased mass transfer, lower surface tension, and higher solubility of numerous compounds [47]. The extraction yield of *S. japonica* increased with an increase in temperature from 180 °C to 420 °C [47], while the *N. zanardinii* extraction yield increased when the extraction time was increased from 10 to 30 min [44]. Similarly, the %PS from *S. japonica* was enhanced by increasing the pressure from 20 to 80 bar [46] and from 13 to 520 bar [47]. Regarding the results obtained in this research, the optimal PLE parameters that will result in the highest PS extraction from *F. virsoides* and *C. barbata* are 0.1 M H_2_SO_4_, for two cycles of 15 min at 140 °C. A temperature of 150 °C, time of 29 min, and solvent to sample ratio of 21 mL g^−1^ were determined to be the optimal conditions for fucoidan extraction from *N. zanardinii* [44], while the optimal conditions for PS extraction from *S. japonica* were a temperature of 127.01 °C, pressure of 80 bar, and sample to solvent ratio of 0.04 g mL^−1^ [46].

*F. virsoides* had a higher total sugar and fucose content than *C. barbata,* which is aligned with the MAE results, while the sulfate group content in PLE was higher in *F.virsoides*. The sulfate group content was higher with acid, which is in accordance with the results for MAE, while a higher fucose and total sugar content were obtained with water, which is reversed from the experiment with MAE. However, the same trend for the fucose content was observed in the fucoidan from *U. pinnatifida* [48] and *Sargassum* sp. [43] because of to the possible breakage of chemical bonds between the fucose structures caused by acid [48].

### 3.3. Comparison of Different Extraction Methods

Table 4 shows the yields and chemical composition of the polysaccharides extracted from *F. virsoides* and *C. barbata*, by conventional and advanced extraction techniques performed under determined optimal conditions. The extraction technique had a significant influence (*p* ≤ 0.05) on the %PS for both algae. Along with a reduced extraction time, from 3 h to 30 min (2 cycles of 15 min), the PLE resulted in a significantly higher %PS from *F. virsoides,* while for *C. barbata,* there was no statistical difference between CE and PLE. Even though MAE was only 10 min, there was no statistical difference (*p* ≤ 0.05) in %PS between the CE and MAE for both algae, meaning that a similar yield was achieved in a much shorter time. Compared with the CE techniques, applying PLE resulted in a significantly higher PS% from *N. zanardinii* [44] and *S. japonica* [13,46], while CE was more efficient than MAE from *A. nodosum* [14,49]. The *N. zanardinii* %PS obtained by PLE (water; 1500 W; 150 °C; two cycles of 10 min) was 13.15 ± 1.05%, which is significantly higher than the 6.17 ± 0.62% and 5.2 ± 0.5% obtained by MAE and conventional hot water extraction, respectively [45]. Under a high temperature and pressure, the physical properties of a solvent are modified, resulting in improved cell destruction, capillary effects, mass transfer, and solvent penetration, and consequentially in increased extraction yields [50].

While the highest total sugar content in *F. virsoides* was obtained by CE and the lowest by MAE, in *C. barbata,* the total sugar content obtained by PLE and MAE was not statistically different from that of CE. Similar to our results, Alboofetileh et al. [45] achieved the lowest total sugar content with MAE, followed by PLE and CE. On the contrary, a lower total sugar content was obtained for CE than for MAE [49] and PLE [44,46]. The *F. virsoides* fucose content was higher with both of the applied advanced techniques, while the *C. barbata* fucose content was not significantly influenced (*p* ≥ 0.05) by the extraction technique. Comparable results were reported for *N. zanardinii* polysaccharides, where the extract obtained by PLE had a higher fucose content than for MAE and CE. Likewise, a higher fucose content was obtained by MAE [49] and PLE in comparison with CE [46]. The sulfate group content for both algae was the highest with PLE, followed by MAE and CE. In the research by Alboofetileh et al. [45], the higher sulfate group content was achieved with MAE and the lowest with PLE. However, it has been reported that MAE resulted in a lower sulfate group content than CE [14,49], while the sulfate group content was lower for PLE in the research by Alboofetileh et al. [44], but higher for Sivagnanam Saravana et al. [46]. A higher PS sulfate group content is an advantageous property, as it has been reported that PS with a higher sulfate content display a higher biological activity. Therefore, the increased sulfate group content observed during these advanced extraction techniques could potentially increase the antioxidant, anticoagulant and anti-HIV (human immunodeficiency virus) activity of extracted PS [14]. For both algae, CE resulted in the highest uronic acid content, while the application of PLE led to a lower uronic acid content, which is opposite to the results obtained for *N. zanardinii,* where the uronic acid content was the highest in the extract obtained by PLE, while there was no statistical difference between MAE and CE [45]. In comparison with CE, a higher uronic acid content was reported by PLE for fucoidan from *S. japonica* [46], as well as MAE for *A. nodosum* [14,49]. On the other hand, *N. zanardinii* fucoidan contained 2.07% uronic acid for PLE and 3.9% for CE [44].

The monosaccharide compositions of the CE, MAE, and PLE extracted fucoidans from *C. barbata* and *F.virsoides* are shown in Table 5. In all of the extracted fucoidans, L-fucose was the predominant monosaccharide, which was expected and in accordance with most of the previously published studies [45,51,52,53,54]. Other detected monosaccharides were glucose, galacturonic acid (oxidized form of D-galactose), and arabinose, while mannose, rhamnose, and fructose were not detected. Glucose and galactose were detected in the majority of similar studies, but in different ratios, e.g., Foley et al. [53] reported 21.3 mol% glucose and 6.1 mol% galactose in *A. nodosum* fucoidan, while Wang et al. [52] reported 1.93 mol% glucose and 24.33 mol% galactose in *Laminaria japonica*. This study also confirmed that the monosaccharides ratio varies according to the extraction method used, which was previously observed by Alboofetileh et al. [45].

The molecular properties of the PS extracts (Table 5) were analyzed using HPSEC, which provided information on the weight average molecular weight (M_w_), the number average molecular weight (M_n_), and the polydispersity index (PDI). M_n_ is the statistical average molecular weight of all of the polymer chains within a sample, whereas M_w_ represents the molecular size of the sample [55]. M_w_ is more influenced by high molecular weight chains, while M_n_ is more influenced by lower molecular weight chains [55]. PDI is the ratio between M_w_ and M_n_ and it measures the heterogeneity of the molecular weight distributions of polymers, where a larger difference between M_w_ and M_n_ (larger PDI) indicates a more heterogeneous molecular weight distribution [55].

The M_w_ of CE, MAE, and PLE extracted PS ranged between 521.72 to 891.25 kDa and 766 to 1252.19 kDa for *F.virsoides* and *C. barbata,* respectively, and fell within the range of reported fucoidan M_w_ values, namely 1.4–1323 kDa [14,26]. The differences in the molecular weight, when compared with the literature values, could be attributed to the algal species and growth conditions, and it is highly dependent on the extraction methodology used [55]. According to their molecular weight, fucoidans can be classified as low-molecular-weight fucoidans (<10 kDa), medium-molecular-weight fucoidans (10–10,000 kDa), and high-molecular-weight fucoidans (>10,000 kDa) [56]. For both algae, the highest M_w_ was achieved in the samples obtained by MAE, while the lowest M_w_ for *F. virsoides* was in the PLE samples and for *C. barbata* it was in the CE samples. Likewise, the *N. zanardinii* M_w_ obtained by MAE was the highest (1184 kDa), followed by CE (823 kDa), while a significantly lower M*w* was in the extract obtained by PLE (670 kDa) [45]. On the contrary, the M_w_ of the *A. nodosum* fucoidan extracted by MAE (30.8 kDa) was significantly lower compared with the samples obtained by CE (40.2 kDa), ultrasound-assisted extraction (121.1 kDa), and enzyme-assisted extraction (100.1 kDa) [49], as microwave heating contributed to the cell wall degradation and splitting of the poly-/oligo-saccharides in the extraction medium [49].

Natural polymers such as proteins are usually monodisperse with a PDI of approximately 1, while polysaccharides are polydisperse with PDIs higher than 1. The PDI of the PS extracted in this study ranged from 1.84 to 3.49, and it fell within the 1 to 6.2 [31,45] range of reported fucoidan PDI values. For both algae, the PDI was the highest in the samples obtained by PLE, indicating a larger degradation during the extraction process. Likewise, PDI was lower in the *N. zanardinii* fucoidan extracted with CE (1.56) compared with MAE (1.84) and PLE (1.78) [45].

**Figure 1:**
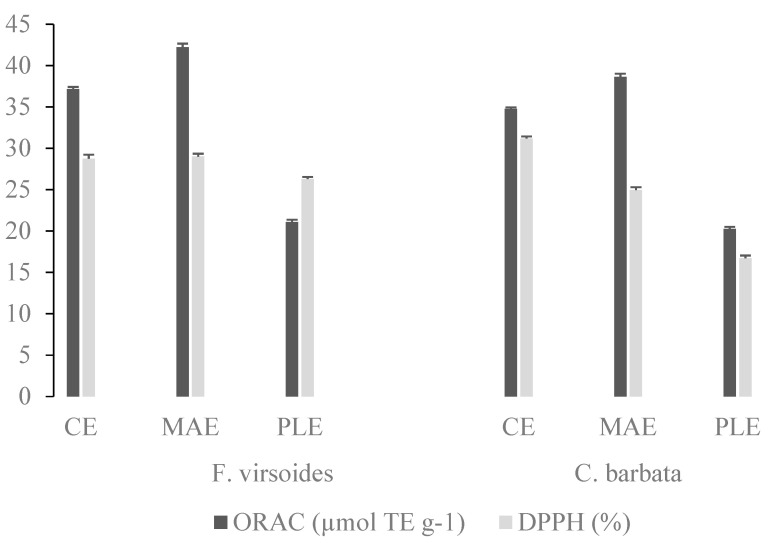
Antioxidant capacity of *F. virsoides* and *C. barbata* polysaccharides, obtained by conventional extraction (CE), microwave assisted extraction (MAE), and pressurized liquid extraction (PLE), determined by oxygen radical absorbance capacity (ORAC) and DPPH assays.

In order to determine the antioxidant capacity of the PS extracts obtained at optimized conditions for each method, ORAC and DPPH assays were employed and the results are shown in Figure 1. The PS from *F. virsoides* and *C. barbata* obtained with MAE had the highest ORAC value of 42.22 ± 0.12 and 38.62 ± 0.12 µmol TE g^−1^, respectively. Furthermore, the extracts obtained with PLE had the lowest ORAC and DPPH values for both algae, which is possibly linked with their chemical structure (higher sulfate content, lower uronic acid content, and lower M_w_). Even though the antioxidant activity of fucoidan has been previously confirmed, the relationship between chemical structure and antioxidant activity, up until now, has not been established. It is known that the antioxidant activity is not a function of a single factor, but a combination of several related physicochemical characteristics, such as the uronic acid content, sulfate group content, protein content, and molecular weight [34]. Therefore, we checked if there was a correlation between the structural characteristics (uronic acid, sulfate group, M_w_, and PDI) and antioxidant measurements (ORAC and DPPH values). Sulfate group content had a significant (*p* ≤ 0.05) strong positive correlation (r > 0.8) with the ORAC and DPPH values, while the uronic acid content, M_w_, and PDI did not correlate with the ORAC and DPPH values. The ORAC values were not well correlated with the total sulfate content and molecular weight of red algae *Gigartina skottsbergii* and *Schizymenia binderi* and brown algae *Lessonia vadosa* [57].

## 4. Conclusions

Advanced extraction techniques, namely, MAE and PLE, were successfully applied and optimized to extract sulfated polysaccharides from brown algae *F. virsoides* and *C. barbata*. PLE under optimal extraction parameters (0.1 M H_2_SO_4_, for two cycles of 15 min at 140 °C) resulted in a significantly higher %PS from *F. virsoides,* while for *C. barbata,* a similar yield was achieved with CE and PLE. Likewise, a similar yield was achieved with MAE (0.1 M H_2_SO_4_ for 10 min at 80 °C) and CE for both algae. Although advanced extraction techniques did not excessively improve the polysaccharide yield, the extraction time was reduced from 3 h to 30 min (PLE) or 10 min (MAE), which contributed to significant energy saving. Furthermore, the polysaccharides obtained by PLE had the highest PDI, fucose, and sulfate group content, and the lowest uronic acid content; however, the antioxidant activity was lower. The correlation between the chemical structure and biological activity, as one of the emerging questions in this field, still remains unclear. These findings indicate that PLE and MAE could be effectively used as a potential method for polysaccharide extraction from brown seaweed, while their apparent antioxidant activity makes these polysaccharides interesting for use in processed and functional food, and in the pharmaceutical and chemical industries.

## Figures and Tables

**Table 1:** Calibration curves for the 1-phenyl-3-methyl-5-pyrazolone (PMP) sugars.

PMP Sugar	Standard Curve	R^2^
arabinose	y = 1.2784x − 0.2645	0.9948
glucose	y = 2.8327x − 0.6781	0.9925
fucose	y = 0.3549x − 0.0712	0.9940
galacturonic acid	y = 2.3517x − 0.6414	0.9951
rhamnose	y = 0.0706x + 0.0064	0.9961
fructose	y = 0.1633x − 0.0453	0.9925

**Table 2:** Influence of algae species (*F. virsoides* and *C. barbata*), extraction solvent (water, 0.1 M HCl, and 0.1 M H_2_SO_4_), time (10, 20, and 30 min), and temperature (60, 80, 100 °C) in microwave assisted extraction on the yield (%PS) and chemical composition of the extracted polysaccharides.

	N	% PS	Total Sugar (%)	Fucose (%)	Sulfate Group (%)
Algae		*p* ≤ 0.01 ^†^	*p* ≤ 0.01 ^†^	*p* ≤ 0.01 ^†^	*p* ≤ 0.01 ^†^
*F. virsoides*	54	13.19 ± 0.02 ^b^	15.40 ± 0.12 ^b^	58.55 ± 0.44 ^b^	25.60 ± 0.25 ^a^
*C. barbata*	54	6.43 ± 0.02 ^a^	6.37 ± 0.12 ^a^	26.13 ± 0.44 ^a^	34.80 ± 0.25 ^b^
Solvent		*p* ≤ 0.01 ^†^	*p* ≤ 0.01 ^†^	*p* ≤ 0.01 ^†^	*p* ≤ 0.01 ^†^
H_2_O	36	5.87 ± 0.03 ^a^	9.07 ± 0.14 ^a^	33.49 ± 0.54 ^a^	19.26 ± 0.31 ^a^
0.1 M HCl	36	8.14 ± 0.03 ^b^	13.20 ± 0.14 ^b^	50.67 ± 0.54 ^c^	25.11 ± 0.31 ^b^
0.1 M H_2_SO_4_	36	15.43 ± 0.03 ^c^	10.39 ± 0.14 ^a^	42.86 ± 0.54 ^b^	46.22 ± 0.31 ^c^
Time (min)		*p* ≤ 0.01 ^†^	*p* ≤ 0.01 ^†^	*p* ≤ 0.01 ^†^	*p* ≤ 0.05 ^†^
10	36	9.91 ± 0.03 ^b^	10.35 ± 0.14 ^a^	43.67 ± 0.53 ^b^	30.02 ± 0.31 ^a,b^
20	36	9.79 ± 0.03 ^a^	10.58 ± 0.14 ^a^	42.16 ± 0.53 ^a,b^	30.89 ± 0.31 ^b^
30	36	9.74 ± 0.03 ^a^	11.72 ± 0.14 ^b^	41.19 ± 0.53 ^a^	29.68 ± 0.31 ^a^
Temperature (°C)		*p* ≤ 0.01 ^†^	*p* ≤ 0.01 ^†^	*p* = *0*.34 ^‡^	*p* ≤ 0.05 ^†^
60	36	8.54 ± 0.03 ^a^	9.39 ± 0.14 ^a^	42.43 ± 0.54 ^a^	31.59 ± 0.31 ^b^
80	36	10.30 ± 0.03 ^b^	11.69 ± 0.14 ^b^	42.85 ± 0.54 ^a^	31.26 ± 0.31 ^b^
100	36	10.60 ± 0.03 ^b^	11.58 ± 0.14 ^b^	41.74 ± 0.54 ^a^	27.75 ± 0.31 ^a^
Algae; solvent		*p* ≤ 0.01 ^†^	*p* ≤ 0.01 ^†^	*p* ≤ 0.01 ^†^	*p* ≤ 0.01 ^†^
*F. virsoides*; H_2_O	18	8.30 ± 0.04 ^b^	14.22 ± 0.20 ^c^	47.30 ± 0.76 ^c^	18.77 ± 0.43 ^a^
*F. virsoides*; 0.1 M HCl	18	12.93 ± 0.04 ^c^	19.95 ± 0.20 ^d^	72.60 ± 0.76 ^e^	21.71 ± 0.43 ^a^
*F. virsoides*; 0.1 M H_2_SO_4_	18	18.35 ± 0.04 ^d^	12.04 ± 0.20 ^c^	55.76 ± 0.76 ^d^	36.30 ± 0.43 ^c^
*C. barbata*; H_2_O	18	3.43 ± 0.04 ^a^	3.92 ± 0.20 ^a^	19.69 ± 0.76 ^a^	19.76 ± 0.43 ^a^
*C. barbata*; 0.1 M HCl	18	3.35 ± 0.04 ^a^	6.45 ± 0.20 ^b^	28.73 ± 0.76 ^b^	28.51 ± 0.43 ^b^
*C. barbata*; 0.1 M H_2_SO_4_	18	12.51 ± 0.04 ^c^	8.74 ± 0.20 ^b^	29.96 ± 0.76 ^b^	56.13 ± 0.43 ^d^
Algae; time (min)		*p* ≤ 0.01 ^†^	*p* ≤ 0.01 ^†^	*p* ≤ 0.01 ^†^	*p* = 16 ^‡^
*F. virsoides*; 10	18	13.85 ± 0.04 ^d^	15.24 ± 0.20 ^c^	62.23 ± 0.76 ^d^	26.17 ± 0.43 ^a^
*F. virsoides*; 20	18	12.67 ± 0.04 ^c^	14.68 ± 0.20 ^c^	55.86 ± 0.76 ^c^	25.11 ± 0.43 ^a^
*F. virsoides*; 30	18	13.06 ± 0.04 ^c,d^	16.29 ± 0.20 ^d^	57.57 ± 0.76 ^c^	25.51 ± 0.43 ^a^
*C. barbata*; 10	18	5.96 ± 0.04 ^a^	5.48 ± 0.20 ^a^	25.11 ± 0.76 ^a^	33.88 ± 0.43 ^b^
*C. barbata*; 20	18	6.91 ± 0.04 ^b^	6.48 ± 0.20 ^b^	28.45 ± 0.76 ^b^	36.67 ± 0.43 ^c^
*C. barbata*; 30	18	6.42 ± 0.04 ^a,b^	7.16 ± 0.20 ^b^	24.82 ± 0.76 ^a^	33.85 ± 0.43 ^b^
Algae; temperature (°C)		*p* ≤ 0.01 ^†^	*p* ≤ 0.01 ^†^	*p* ≤ 0.01 ^†^	*p* ≤ 0.01 ^†^
*F. virsoides*; 60	18	11.72 ± 0.04 ^d^	13.78 ± 0.20 ^d^	61.51 ± 0.76 ^c^	26.46 ± 0.43 ^b^
*F. virsoides*; 80	18	14.36 ± 0.04 ^f^	16.85 ± 0.20 ^f^	60.51 ± 0.76 ^c^	27.41 ± 0.43 ^b^
*F. virsoides*; 100	18	13.50 ± 0.04 ^e^	15.58 ± 0.20 ^e^	53.64 ± 0.76 ^b^	22.92 ± 0.43 ^a^
*C. barbata*; 60	18	5.37 ± 0.04 ^a^	5.00 ± 0.20 ^a^	23.34 ± 0.76 ^a^	36.72 ± 0.43 ^d^
*C. barbata*; 80	18	6.24 ± 0.04 ^b^	6.53 ± 0.20 ^b^	25.20 ± 0.76 ^a^	35.10 ± 0.43 ^d^
*C. barbata*; 100	18	6.69 ± 0.04 ^c^	7.59 ± 0.20 ^c^	29.84 ± 0.76 ^a^	32.58 ± 0.43 ^c^

Values with different letters are statistically different at *p* ≤ 0.05. ^†^ Statistically significant variables at *p* ≤ 0.05. ^‡^ Statistically insignificant variables at *p* ≤ 0.05.

**Table 3:** Influence of algae species (*F. virsoides* and *C. barbata*), extraction solvent (water and 0.1 M H_2_SO_4_), temperature (60, 100, and 140 °C), time (5, 10, and 15 min), and number of cycles (1 and 2) in pressurized liquid extraction on the yield (%PS) and chemical composition of the extracted polysaccharides.

	N	% PS	Total Sugar (%)	Fucose (%)	Sulfate Group (%)
Algae		*p* ≤ 0.01 ^†^	*p* ≤ 0.01 ^†^	*p* ≤ 0.01 ^†^	*p* ≤ 0.01 ^†^
*F. virsoides*	72	10.22 ± 0.03 ^a^	14.50 ± 0.09 ^b^	42.03 ± 0.19 ^b^	65.70 ± 0.42 ^b^
*C. barbata*	72	11.77 ± 0.03 ^b^	7.83 ± 0.09 ^a^	13.57 ± 0.19 ^a^	60.45 ± 0.42 ^a^
Solvent		*p* ≤ 0.01 ^†^	*p* ≤ 0.01 ^†^	*p* ≤ 0.01 ^†^	*p* ≤ 0.01 ^†^
H_2_O	72	5.07 ± 0.03 ^a^	17.19 ± 0.09 ^b^	33.18 ± 0.19 ^b^	58.32 ± 0.42 ^a^
0.1 M H_2_SO_4_	72	16.93 ± 0.03 ^b^	5.14 ± 0.09 ^a^	22.41 ± 0.19 ^a^	67.83 ± 0.42 ^b^
Temperature (°C)		*p* ≤ 0.01 ^†^	*p* ≤ 0.01 ^†^	*p* ≤ 0.05 ^†^	*p* ≤ 0.01 ^†^
60	48	8.39 ± 0.04 ^a^	11.86 ± 0.11 ^c^	27.24 ± 0.24 ^a^	75.42 ± 0.52 ^b^
100	48	10.96 ± 0.04 ^b^	11.17 ± 0.11 ^b^	28.02 ± 0.24 ^a,b^	57.26 ± 0.52 ^a^
140	48	13.64 ± 0.04 ^c^	10.48 ± 0.11 ^a^	28.13 ± 0.24 ^b^	56.55 ± 0.52 ^a^
No. of cycles		*p* ≤ 0.01 ^†^	*p* ≤ 0.01 ^†^	*p* ≤ 0.01 ^†^	*p* ≤ 0.01 ^†^
1	72	10.67 ± 0.03 ^a^	11.70 ± 0.09 ^b^	28.54 ± 0.19 ^b^	64.86 ± 0.42 ^b^
2	72	11.33 ± 0.03 ^b^	10.64 ± 0.09 ^a^	27.05 ± 0.19 ^a^	61.29 ± 0.42 ^a^
Time (min)		*p* ≤ 0.01 ^†^	*p* ≤ 0.01 ^†^	*p* ≤ 0.01 ^†^	*p* ≤ 0.01 ^†^
5	48	9.94 ± 0.04 ^a^	12.37 ± 0.11 ^b^	28.48 ± 0.24 ^b^	62.29 ± 0.52 ^a^
10	48	11.21 ± 0.04 ^b^	10.49 ± 0.11 ^a^	26.22 ± 0.24 ^a^	60.64 ± 0.52 ^a^
15	48	11.84 ± 0.04 ^c^	10.65 ± 0.11 ^a^	28.69 ± 0.24 ^b^	66.30 ± 0.52 ^b^
Algae; solvent		*p* ≤ 0.01 ^†^	*p* ≤ 0.01 ^†^	*p* ≤ 0.01 ^†^	*p* ≤ 0.01 ^†^
*F. virsoides*; H_2_O	36	6.40 ± 0.05 ^b^	22.03 ± 0.13 ^d^	54.85 ± 0.27 ^d^	63.48 ± 0.60 ^b^
*F. virsoides*; 0.1 M H_2_SO_4_	36	14.04 ± 0.05 ^c^	6.98 ± 0.13 ^b^	29.20 ± 0.27 ^c^	67.93 ± 0.60 ^c^
*C. barbata*; H_2_O	36	3.73 ± 0.05 ^a^	12.36 ± 0.13 ^c^	11.51 ± 0.27 ^a^	53.17 ± 0.60 ^a^
*C. barbata*; 0.1 M H_2_SO_4_	36	19.81 ± 0.05 ^d^	3.31 ± 0.13 ^a^	15.62 ± 0.27 ^b^	67.73 ± 0.60 ^c^
Algae; temperature (°C)		*p* ≤ 0.01 ^†^	*p* ≤ 0.01 ^†^	*p* ≤ 0.01 ^†^	*p* ≤ 0.01 ^†^
*F. virsoides*; 60	24	7.10 ± 0.06 ^a^	15.49 ± 0.16 ^d^	45.17 ± 0.33 ^d^	80.52 ± 0.73 ^d^
*F. virsoides*; 100	24	9.17 ± 0.06 ^b^	14.21 ± 0.16 ^c^	39.99 ± 0.33 ^c^	58.12 ± 0.73 ^b^
*F. virsoides*; 140	24	14.40 ± 0.06 ^e^	13.82 ± 0.16 ^c^	40.92 ± 0.33 ^c^	58.46 ± 0.73 ^b^
*C. barbata*; 60	24	9.68 ± 0.06 ^c^	8.22 ± 0.16 ^b^	9.31 ± 0.33 ^a^	70.32 ± 0.73 ^c^
*C. barbata*; 100	24	12.75 ± 0.06 ^d^	8.14 ± 0.16 ^b^	16.05 ± 0.33 ^b^	56.40 ± 0.73 ^a,b^
*C. barbata*; 140	24	12.89 ± 0.06 ^d^	7.13 ± 0.16 ^a^	15.34 ± 0.33 ^b^	54.63 ± 0.73 ^a^
Algae; no. of cycle		*p* ≤ 0.01 ^†^	*p* ≤ 0.01 ^†^	*p* ≤ 0.01 ^†^	*p* ≤ 0.01 ^†^
*F. virsoides*; 1	36	10.02 ± 0.05 ^a^	15.49 ± 0.13 ^a^	44.32 ± 0.27 ^d^	65.13 ± 0.60 ^b^
*F. virsoides*; 2	36	10.43 ± 0.05 ^b^	13.52 ± 0.13 ^b^	39.74 ± 0.27 ^c^	66.27 ± 0.60 ^b^
*C. barbata*; 1	36	11.31 ± 0.05 ^c^	7.91 ± 0.13 ^a^	12.76 ± 0.27 ^a^	64.59 ± 0.60 ^b^
*C. barbata*; 2	36	12.23 ± 0.05 ^d^	7.76 ± 0.13 ^a^	14.37 ± 0.27 ^b^	56.31 ± 0.60 ^a^
Algae; time (min)		*p* ≤ 0.01 ^†^	*p* ≤ 0.01 ^†^	*p* ≤ 0.01 ^†^	*p* ≤ 0.01 ^†^
*F. virsoides*; 5	24	8.47 ± 0.06 ^a^	16.04 ± 0.16 ^e^	44.24 ± 0.33 ^e^	63.54 ± 0.73 ^b,c^
*F. virsoides*; 10	24	10.33 ± 0.06 ^b^	13.92 ± 0.16 ^d^	39.22 ± 0.33 ^c^	67.90 ± 0.73 ^d^
*F. virsoides*; 15	24	11.87 ± 0.06 ^d,e^	13.55 ± 0.16 ^d^	42.63 ± 0.33 ^d^	65.66 ± 0.73 ^c,d^
*C. barbata*; 5	24	11.41 ± 0.06 ^c^	8.69 ± 0.16 ^c^	12.71 ± 0.33 ^a^	61.03 ± 0.73 ^b^
*C. barbata*; 10	24	12.10 ± 0.06 ^e^	7.06 ± 0.16 ^a^	13.23 ± 0.33 ^a^	53.38 ± 0.73 ^a^
*C. barbata*; 15	24	11.80 ± 0.06 ^d^	7.74 ± 0.16 ^b^	14.75 ± 0.33 ^b^	66.95 ± 0.73 ^d^

Values with different letters are statistically different at *p* ≤ 0.05. ^†^ Statistically significant variables at *p* ≤ 0.05.

**Table 4:** Yield (% PS) and chemical composition of CE (conventional), MAE (microwave) and PLE (pressurised liquid) extracted polysaccharides from *Fucus virsoides* and *Cystoseira barbata*.

		% PS	Total Sugar (%)	Fucose (%)	Sulfate Group (%)	Uronic Acid (%)
*F. virsoides*		*p* ≤ 0.05 ^†^	*p* ≤ 0.05 ^†^	*p* ≤ 0.05 ^†^	*p* ≤ 0.05 ^†^	*p* ≤ 0.05 ^†^
	CE	18.53 ± 0.00 ^a^	20.17 ± 0.00 ^c^	41.54 ± 0.01 ^a^	28.46 ± 0.01 ^a^	20.06 ± 0.00 ^c^
	MAE	20.42 ± 0.28 ^a^	15.65 ± 0.09 ^a^	48.48 ± 1.36 ^b^	37.13 ± 0.26 ^b^	15.93 ± 0.77 ^b^
	PLE	24.22 ± 0.94 ^b^	18.24 ± 0.13 ^b^	60.08 ± 0.22 ^c^	51.82 ± 1.72 ^c^	5.32 ± 0.51 ^a^
*C. barbata*		*p* ≤ 0.05 ^†^	*p* ≤ 0.05 ^†^	*p* = 0.11 ^‡^	*p* ≤ 0.05 ^†^	*p* ≤ 0.05 ^†^
	CE	16.47 ± 0.18 ^a,b^	6.34 ± 0.18 ^a,b^	22.53 ± 0.14 ^a^	35.53 ± 0.80 ^a^	15.72 ± 0.34 ^c^
	MAE	15.27 ± 0.17 ^a^	7.14 ± 0,53 ^b^	26.61 ± 2.15 ^a^	45.56 ± 0.34 ^b^	12.52 ± 0.08 ^b^
	PLE	18.77 ± 0.82 ^b^	4.40 ± 0.19 ^a^	28.06 ± 0.44 ^a^	57.58 ± 2.19 ^c^	7.15 ± 0.36 ^a^

Values with different letters are statistically different at *p* ≤ 0.05. ^†^ Statistically significant variable at *p* ≤ 0.05. ^‡^ Statistically insignificant variable at *p* ≤ 0.05.

**Table 5:** Monosaccharide composition and molecular properties (weight average molecular weight—M_w_; number average molecular weight—M_n_; polydispersity index—PDI) of CE (conventional), MAE (microwave), and PLE (pressurized liquid) extracted polysaccharides from *Fucus virsoides* and *Cystoseira barbata*.

		Monosacharide Composition (%)				Molecular Properties		
		Glucose	Fucose	Galacturonic Acid	Arabinose	M_w_ (kDa)	M_n_ (kDa)	Polydispersity (M_w_/M_n_)
*F. virsoides*		*p* ≤ 0.05 ^†^	*p* ≤ 0.05 ^†^	*p* ≤ 0.05 ^†^	*p* ≤ 0.05 ^†^			
	CE	18.65 ± 0.24 ^b^	44.83 ± 0.45 ^b^	19.48 ± 0.26 ^b^	17.04 ± 0.25 ^c^	693.43	264.42	2.62
	MAE	13.26 ± 0.36 ^a^	78.35 ± 0.21 ^c^	n.d. ^a^	8.39 ± 0.19 ^a^	891.25	332.14	2.68
	PLE	19.04 ± 0.41 ^b^	41.90 ± 0.33 ^a^	26.52 ± 0.31 ^c^	12.55 ± 0.35 ^b^	521.72	149.64	3.49
*C. barbata*		*p* ≤ 0.05 ^†^	*p* ≤ 0.05 ^†^	*p* ≤ 0.05 ^†^	*p* ≤ 0.05 ^†^			
	CE	n.d.^a^	100 ± 0.00 ^c^	n.d. ^a^	n.d. ^a^	766.00	322.87	2.37
	MAE	27.22 ± 0.16 ^c^	61.27 ± 0.51 ^b^	n.d. ^a^	11.52 ± 0.32 ^b^	1252.19	681.34	1.84
	PLE	16.95 ± 0.38 ^b^	49.5 ± 0.28 ^a^	19.75 ± 0.38 ^b^	13.79 ± 0.48 ^c^	1031.94	415.75	2.48

Values with different letters are statistically different at *p* ≤ 0.05. ^†^ Statistically significant variables at *p* ≤ 0.05.
